# Alteration of Sexual Reproduction and Genetic Diversity in the Kelp Species *Laminaria digitata* at the Southern Limit of Its Range

**DOI:** 10.1371/journal.pone.0102518

**Published:** 2014-07-14

**Authors:** Luz Valeria Oppliger, Peter von Dassow, Sarah Bouchemousse, Marine Robuchon, Myriam Valero, Juan A. Correa, Stéphane Mauger, Christophe Destombe

**Affiliations:** 1 Departamento de Ecología, Facultad de Ciencias Biológicas, Pontificia Universidad Católica de Chile, Santiago, Chile; 2 Sorbonne Universités, UPMC Univ. Paris 06, UMR 7144, Station Biologique de Roscoff, Roscoff, France; 3 CNRS, UMR 7144, Station Biologique de Roscoff, Place Georges Teissier, Roscoff, France; 4 Instituto Milenio de Oceanografía (IMO), Pontificia Universidad Católica de Chile, Santiago, Chile; 5 Institut de Systématique, Evolution, Biodiversité, UMR 7205, Muséum national d’Histoire naturelle, Paris, France; 6 Center of Applied Ecology & Sustainability, Facultad de Ciencias Biológicas, Pontificia Universidad Católica de Chile, Santiago, Chile; College of Charleston, United States of America

## Abstract

Adaptation to marginal habitats at species range-limits has often been associated with parthenogenetic reproduction in terrestrial animals and plants. Laboratory observations have shown that brown algae exhibit a high propensity for parthenogenesis by various mechanisms. The kelp *Laminaria digitata* is an important component of the ecosystem in Northern European rocky intertidal habitats. We studied four *L. digitata* populations for the effects of marginality on genetic diversity and sexual reproduction. Two populations were marginal: One (Locquirec, in Northern Brittany) was well within the geographic range, but was genetically isolated from other populations by large stretches of sandy beaches. Another population was at the range limits of the species (Quiberon, in Southern Brittany) and was exposed to much higher seasonal temperature changes. Microsatellite analyses confirmed that these populations showed decreased genetic and allelic diversity, consistent with marginality and genetic isolation. Sporophytes from both marginal populations showed greatly diminished spore-production compared to central populations, but only the southern-limit population (Quiberon) showed a high propensity for producing unreduced (2N) spores. Unreduced 2N spores formed phenotypically normal gametophytes with nuclear area consistent with ≥2N DNA contents, and microsatellite studies suggested these were produced at least in part by automixis. However, despite this being the dominant path of spore production in Quiberon sporophyte individuals, the genetic evidence indicated the population was maintained mostly by sexual reproduction. Thus, although spore production and development showed the expected tendency of geographical parthenogenesis in marginal populations, this appeared to be a consequence of maladaptation, rather than an adaptation to, life in a marginal habitat.

## Introduction

The ubiquity of sex among eukaryotes has long been a paradox due to the high costs associated with mating and recombination [1], [2]. Sex incurs both significant genetic costs, as parents share only half their genes with their progeny, and energetic costs, as meiosis is slower than mitosis and more prone to failure, and sexual reproduction also requires investment of energy into encountering compatible mates. Theoretically, asexual females transmit twice as many genes to each offspring compared to sexually reproducing females [1], yet sex is the predominant mode of reproduction in nearly all multicellular taxa. Several theories attempt to explain the evolutionary maintenance of sex through the benefits of genetic reshuffling which accelerates the production of advantageous new genotypes, facilitating adaptation, and limiting the accumulation of deleterious mutations [3], [4], [5], [6], [7]. Sexual and asexual lineages may co-exist in the long term as a result of a dynamic equilibrium between the origin of new asexual lineages and their extinction [8].

Geographical parthenogenesis refers to the case where closely related sexual and asexual lineages exhibit distinct distributions [9]. Asexual forms often tend to be prevalent in populations that occupy the margins of a species range, including high altitudes, deserts, or small islands. Geographical parthenogenesis has been observed in several organisms including both marine and terrestrial arthropods [10], [11], vertebrates [12], [13], plants [14], [15] and both red [16], [17], [18] and brown macroalgae [19]. Marginal populations are generally characterized by increased genetic isolation, genetic differentiation, and variability in individual and population performance. Several mechanisms have been proposed to explain these patterns (reviewed in [20]), including: 1. Sexuality may be more advantageous in habitats when selection results from biotic interactions, as a co-evolutionary arms-race with parasites, predators and competitors favor continued generation of new gene combinations [21], [22]. In contrast, asexuality might be favored in sparsely inhabited regions where abiotic factors dominate and the relative energetic costs of mating are higher [23]. 2. Asexuals are superior colonists of new habitats because they do not have the two-fold cost of sex [24]. 3. Asexuality maintains locally adapted gene combinations [25]. 4. Asexuals avoid the cost of inbreeding depression [26].

Brown algae (Phaeophyceae) are a closely-related group of multicellular organisms that form essential structural components of near-shore marine ecosystems. These organisms exhibit a complex haplo-diplontic life cycle where both haploid and diploid phases exhibit vegetative growth. Diploid sporophytes produce spores by meiosis, which then undergo mitotic division to form multicellular haploid gametophytes. Fertilization eventually restores the diploid sporophyte state, which again grows vegetatively. Multiple mechanisms to avoid or modify sexuality exist within brown algae. Haploid cells from the gametophyte can undergo endomitosis to restore diploidy without fertilization in *Ectocarpus* sp., *Laminaria japonica*, *Undaria pinnatifida and Lessonia nigrescens* [27], [28], [29]. Apomeiosis, the replacement of meiosis with a mitotic division to produce diploid spores, has been documented in *Ectocarpus* sp. [29]. Finally, almost all brown algae are capable of reproducing by fragmentation of either haploid or diploid multicellular phases, completely skipping the sexual cycle [30], [31], [32].

The kelp *Laminaria digitata* exhibits a broad distribution along the European coast, with the southern limit clearly defined on the Atlantic coast of Southern Brittany [33]. The present study aims at understanding the reproductive systems of populations at both the center and the edge of distribution of *Laminaria digitata.* We studied four populations for the effects of marginality on genetic diversity and sexual reproduction. Two populations were marginal: One (Locquirec, in Northern Brittany) was well within the geographic range, but was genetically isolated from other populations by large stretches of sandy beaches that represent a local barrier of unfavorable habitat [34], [35], [36]. Another population was at the range limits of the species in Quiberon, in Southern Brittany. Two populations were central: Porspoder and Roscoff (both in central Brittany). We tested the occurrence of geographical parthenogenesis through a combination of distinct approaches including: population genetic analyses, analyses of spore production, flow cytometry and microscopic analyses of spore and gametophyte ploidy, and *in vitro* culture and microscopic observations.

## Materials and Methods

### Site characterization

**Figure 1:**
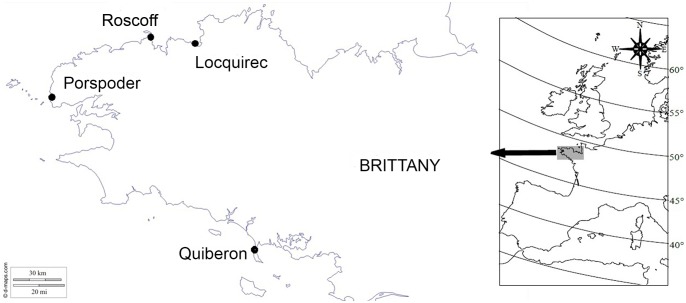
Studied sites of *Laminaria digitata* in Brittany, France.

Two or three sites in each of four regions along the Brittany coast (Locquirec, Roscoff, Porspoder, Quiberon) were selected according their contrasting geographical and ecological characteristics (Figure 1, Table 1).

**Table 1:** Geographic information and summary statistics of the sampled populations.

Region	Site	GPS position	Population type	N	He	Na	AR	*Fis*	R
**Locquirec**	Porz Mellec^1^	48°41′11″N 3°37′06″W	Marginal	19	0.518±0.066	3.71±0.47	3.71±0.47	–0.019±0.045	1.00
	Beg Douar	48°41′12″N 3°36′47″W	Marginal	31	0.519±0.052	4.14±0.55	3.82±0.44	0.033±0.035	1.00
	*Mean*				*0.518*±*0.000*	*3.93*±*0.21*	*3.77*±*0.05*	*0.007*±*0.026*	*1.00*
**Roscoff**	Ar Pourven	48°42′44″N 3°57′30″W	Central	49	0.614±0.070	6.14±1.71	5.31±1.41	0.013±0.030	1.00
	Sieck^1^	48°42′40″N 4°03′37″W	Central	41	0.629±0.070	7.14±1.74	6.03±1.31	–0.019±0.029	1.00
	Duons	48°43′41″N 3°55′32″W	Central	45	0.611±0.076	6.57±1.38	5.29±1.02	0.068±0.041	1.00
	*Mean*				*0.618*±*0.005*	*6.62*±*0.29*	*5.54*±*0.24*	*0.021*±*0.026*	*1.00*
**Porspoder**	St Laurent	48°31′11″N 4°46′45″W	Central	27	0.676±0.046	7.00±1.20	6.44±1.03	–0.011±0.025	1.00
	Le Conquet^2^	48°19′48″N 4°46′25″W	Central	27	0.671±0.061	6.71±1.80	6.22±1.57	0.067±0.048	1.00
	Molène^2^	48°23′49″N 4°56′01″W	Central	20	0.695±0.043	6.43±1.15	6.35±1.14	0.085±0.054	1.00
	*Mean*				*0.681*±*0.007*	*6.71*±*0.17*	*6.33*±*0.07*	*0.047*±*0.030*	*1.00*
**Quiberon**	Pointe ConguelSouth	47°28′09″N 3°05′40″W	Marginal	41	0.495±0.072	4.29±0.94	3.87±0.77	0.071±0.047	0.95
	Pointe deConguel North	47°28′12″N 3°05′29″W	Marginal	49	0.555±0.058	5.43±0.90	4.42±0.68	0.049±0.029	1.00
	Belle-Ile	47°19′41″N 3°07′27″W	Marginal	47	0.474±0.067	4.29±0.61	3.72±0.44	0.027±0.045	0.98
	*Mean*				*0.508*±*0.024*	*4.67*±*0.38*	*4.00*±*0.21*	*0.049*±*0.013*	*0.98*

N is the number of individuals sampled, He is the unbiased genetic diversity [41], Na is the number of alleles, AR is the allelic richness based on the smallest sample size (19 diploid individuals), *Fis* is the departure from random meeting [43] and R is the genotypic diversity ((G−1/N−1, where G is the number of distinct genotypes, [44]). He, Na, AR and *Fis* have been calculated using FSTAT [42]; values by population are mean ± standard error over loci and values by region are mean ± standard error over populations.

1 These sites have already been analyzed in Valero et al. 2011 [35] (Sieck = NB3, Porspoder = NB1 and Porz Mellec = NB4); values might slightly differ because of different sub-sampling in the two analysis.

2 These sites have already been analyzed in Couceiro et al. 2013 [36] (Le Conquet = MPA2 and Molène = MPA1); values might slightly differ because of different sub-sampling in the two analysis.

### Sea surface temperature

Sea surface temperature (SST) data were obtained from daily High Resolution Sea Surface Temperature from 1992 to 2003 over the IBIROOS area (Ireland Biscay Iberia Regional Operating Operational System) in a 0.044° Geographical Grid AVHRR (Advanced Very High Resolution Radiometer) from NOAA [37]. Mean seasonal temperature was estimated for each season: winter (from 15 December to 15 March), spring (from 15 March to 15 June), summer (form 15 June to 15 September), autumn (from 15 September to 15 December) (ECOOP Project: http://www.ecoop.eu.). The full remote sensing dataset for the entire Brittany coast is described in Gallon et al. [38]. Direct data on temperature in the lower intertidal habitat occupied by *L. digitata* sporophytes was directly recorded using temperature data loggers TidbiT v2-UTBI-001 Hobo Onset fixed to rocks in the Roscoff site 48°42′44″N 3°57′30″W between July 2009–March 2010, and in the Quiberon site 47°28′09″N 3°05′40″W between July 2009–January 2010. Data loggers recorded temperature every 15 minutes.

Comparison of mean seasonal temperature between sites was tested using a 2-way ANOVA with season (fixed, 4 levels) and location (fixed, 4 levels) as factors. Differences among locations were considered in relation to season. The response variable was the mean temperature. Data was log transformed in order to fit the assumptions of normality and homogeneity of variances. Type III sum of squares was used for tests of significance. All statistical analyses were done with MINITAB v. 13.2 (State College, PA, USA), by performing ANOVA and Tukey’s tests for *a posteriori* multiple comparisons of means (*α* = 0.05). Comparisons of median temperature between sites recorded from data loggers were made using Mann-Whitney tests MINITAB v. 13.2 (State College, PA, USA).

### Sampling

A total of 135 fertile thalli were sampled along the Brittany coast between Locquirec and Quiberon (Fig. 1) from July to September 2009. No specific permissions were required for these locations/activities. Two locations, Porspoder and Roscoff, were chosen for the continuous distribution of *Laminaria digitata* (hereafter referred to as central populations), and two locations, Quiberon and Locquirec, were sampled, one at the southern range limit of the distribution and the other in northern Brittany. These latter locations were considered to be marginal because they represents the southern-most population of *L. digitata* and the other is isolated by sandy beaches. From 23 to 38 mature sporophytic individuals were collected from these four regions. This sampling was done to analyze spores by flow cytometry and culturing.

In addition, a hierarchical sampling was performed in order to compare genetic diversity within and among the four regions (two sites in Quiberon and 3 sites in Porspoder, Roscoff and Locquirec, Table 1). A total of 396 sporophytes (i.e. 19 to 49 individuals for each sites of the four regions, Table 1) were collected during low spring tides. Tissue samples, consisting of a small disc excised from the base of the blade, were preserved in individual plastic bags containing silica gel and stored at room temperature.

### Sporophyte and gametophyte DNA extraction, PCR and genotyping

For the genetic analyses of the sporophyte individuals, DNA extraction and PCR amplifications of seven microsatellite loci (Ld1-124, Ld2-148, Ld2-158, Ld2-167, Ld2-371, Ld2-531 and Ld2-704) were performed using the same protocols as those described in Billot et al. [39]. In addition, to identify the cytological mechanism that produces diploid spores in *L. digitata*, descendants were genotyped with the two microsatellite markers Ld2-371 and Ld2-531, including 12–24 gametophytes per parent from a total of 8 sporophyte parents. Because of the small amount of material, DNA extractions for gametophytes were done with 5% Chelex 100 (Bio*-*Rad Laboratories, Hercules, CA) and proteinase k 10 mg/mL according to Wattier et al. [40].

PCR products were electrophoresed on 6% polyacrylamide denaturing gels using an automated DNA sequencer (Li-Cor 4200) along with a DNA sequence of known length to estimate allele sizes to ensure that allele sizes corresponded exactly to those estimated in other studies [34], [35], [36].

### Genetic and genotypic diversity analyses

Standard measures of genetic diversity, including mean number of alleles per locus (Na), unbiased expected heterozygosity (He; [41]), and allelic richness (AR) rarefied to 19 individuals for each sites, were computed with the software FSTAT v2.9.3 [42]. To test the hypothesis that central populations were more genetically diverse than marginal populations, differences of He and AR among groups were tested with the software FSTAT v2.9.3 using 5000 random permutations of populations among groups. F_IS_ estimates of the average deviation from random mating within populations [43] were computed for each locus, and heterozygote deficiencies and excesses were tested using 1000 randomizations of alleles among individuals within each population using FSTAT v2.9.3. The number of diploid individual showing the same genotype (repeated multilocus genotypes) by site was calculated using GENALEX 6.2 [44] and the genotype diversity R was computed as the number of distinct genotypes (G)/number of individuals and corrected for sample size (R = (G−1)/(N−1), [45]). In order to estimate the number of repeated multilocus genotype (MLG) and whether these MLGs were produced by sexual or asexual reproduction, GENCLONE 2.0 was used [46]. For each individuals, Psex, which is the probability for a given multilocus genotype to be observed in N samples as a consequence of distinct sexual reproductive events, was calculated for each repeated MLG. If Psex was lower than 0.05, duplicated multilocus genotypes were considered as clones belonging to the same “genet” (i.e., resulting from asexual reproduction).

### Spore release

Fertile fragments of equal size (3.8 cm^2^) were cut off from each plant, cleaned in running tap water and exposed to air for 30 minutes. Ten fragments per plant were transferred to four 50 mL plastic Falcons tubes containing 40 µL of filtered seawater at 0.2 µm, set on ice and agitated. Fertile fragments were withdrawn from the tubes after 12 h. Each spore suspension was divided in three aliquots. The first aliquot was used to estimate the number of released spores per ml by light microscopy with Neubauer chambers, the second one was used to obtain gametophyte cultures, and the third one was fixed with 1% formaldehyde for flow cytometry analyses.

### Flow cytometry analyses

Ploidy levels (relative DNA content) of *L. digitata* spores were estimated by analysis of fixed spores stained with Sybr Green I (Invitrogen, Carlsbad, CA, USA) on a FACSCanto II flow cytometer (BD Biosciences, San Jose, CA, USA). Exponentially growing cultures of haploid *Emiliania huxleyi* RCC1217, a unicellular eukaryotic phytoplankton, were fixed with the same fixative and added as an internal standard during staining. This species was selected as it could be easily distinguished from *L. digitata* spores based on chlorophyll, forward scatter, and side scatter properties. Early trials on isolated nuclei obtained from live spores using previously established protocols [47], [48] found that ploidy analyses of fixed spores and extracted nuclei yielded similar results. However, isolation of nuclei from live spores requires substantial dilution of spores into a nuclear extraction buffer. Analysis of fixed spores was selected to allow consistent analysis of ploidy even from sporophyte thalli that yielded very low spore numbers. Data were analyzed using the BD FACSDiva and FlowJo software (Treestar, Ashland, OR, USA). Spore ploidy was scored as 1N-dominant if the ratio 1N spores/2N spores was ≥3, 2N-dominant the ratio was ≤1/3, and co-dominant if the ratio was between 1/3 and 3.

### Culturing of gametophytes for study of development, sex ratio, ploidy, and meiosis segregation

Five drops of each spore suspension were inoculated in 55 mm x 14 mm plastic Petri dishes with Provasoli enriched seawater and incubated in a culture chamber set at 15°C, 12∶12 light:dark, 25–35 µmol photons m^−2^s^−1^. Culture medium was changed once a week and observations were done monthly for 10 months.

### Sex ratio estimations

Sex ratio estimations were done on 9 progenies from Quiberon. Male and female gametophytes were identified according to their morphological characteristics using a Nikon Eclipse TE300 inverted microscope (Nikon Corp., Tokyo, Japan). Female gametophytes are characterized by large cells and filaments with few branches whereas male gametophytes are smaller and display highly branched filaments formed by small cells. These morphological differences make them unambiguously identifiable under the light microscope. The numbers of male and female gametophytes were determined by counting their occurrence in three visual fields per slide using the 10x objective. Sex ratio was expressed as the frequency of males per progeny (i.e. males/(males+females)).

### Estimation of gametophyte ploidy by microscopy

Comparative ploidy levels of 10 male and female gametophytes from 2 progenies from Quiberon and 2 gametophytes from 2 progenies from Roscoff were estimated by measuring nuclear area using the fluorescent DNA Hoechst stain (Invitrogen, European Headquarters) and epifluorescence inverted microscope Zeiss Observer Z1 (Carl Zeiss Microimaging, Inc., USA), as described in Bothwell et al. (2010). Images of nuclei were analyzed with ImageJ software (National Institutes of Health, available at www.nih.gov).

## Results

### Sea surface temperature between sites

**Figure 2:**
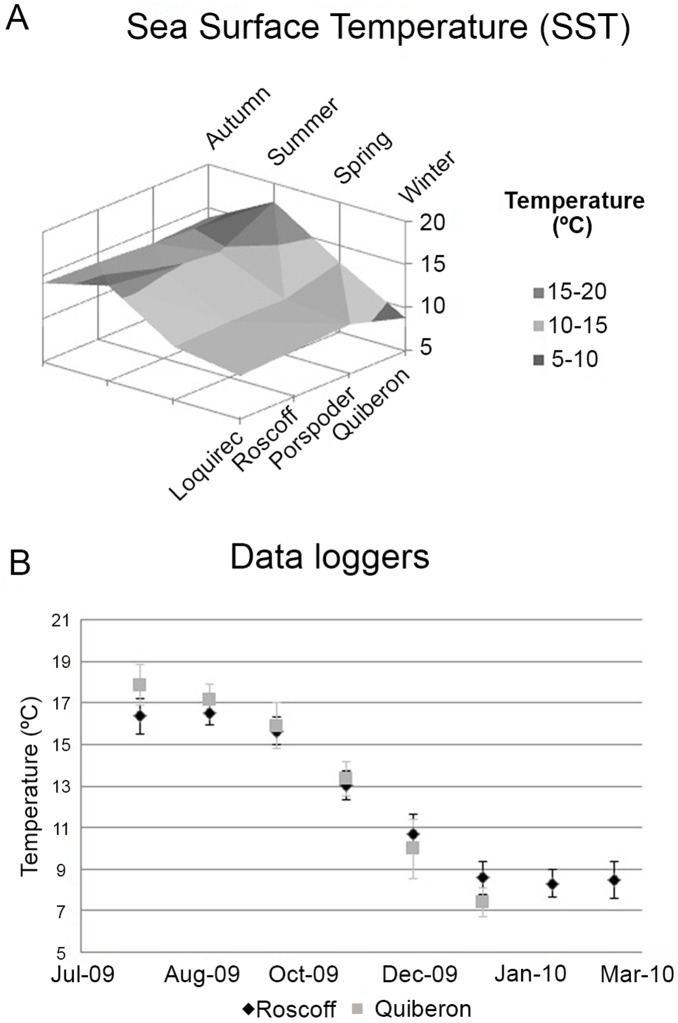
Seasonal temperature variation at Roscoff and Quiberon. A) Seasonal sea surface temperature (SST) determined from satellite (mean seasonal temperature, averaged over all 12 years). B) Direct measurements from data loggers.

Coastal sea surface temperature analysis showed that SST depended on season and location factors (ANOVA, F_3,191_ = 40.37, p<0.0001, F_3,191_ = 804.06, p<0.0001). The season by location interaction was significant (ANOVA F_9,191_ = 19.84, P<0.0001) (Fig. 2A). The Quiberon site experienced higher spring (mean and standard deviation 13.06±0.32) and summer (17.89±0.66) temperature than Roscoff, Porspoder and Locquirec (in spring 11.46±0.51, 11.38±0.44, 11.15±0.49 and in summer 15.20±0.48, 14.64±0.50, 16.00±0.39, respectively). In addition, the mean summer temperature recorded in the Locquirec site was higher than those recorded in Roscoff and Porspoder (Tukey test: p<0.05). On the contrary, the mean winter temperature in Quiberon was lower (8.83±1.03) than in the other sites (10.34±0.528, 10.71±0.42, 9.98±0.47, respectively) (Tukey test: p<0.05). Local data loggers in the lower intertidal confirmed that Quiberon experienced higher summer temperatures and lower winter temperatures than Roscoff (Mann and Whitney test p<0.001) with in August median values of temperature in Quiberon and Roscoff of 17.46 and 16.39 respectively and in January 8.17 and 9.17 respectively) (Fig. 2B).

### Genetic analysis of sporophyte populations

The number of alleles varied from 2 (locus Ld1-124, site Pointe de Conguel, region of Quiberon) to 17 (locus Ld1-371, sites of Sieck and site of Le Conquet, in the two central regions of Roscoff and Porspoder). Microsatellite analysis (Table 1) showed that marginal populations located in the sites in Quiberon and Locquirec showed significantly lower genetic diversity (AR and He) than populations located in the central area (Porspoder and Roscoff) (permutation tests of comparison among central versus marginal populations for He, p-value = 0.002; for AR, p-value = 0.002). The lowest genetic diversity (He) was found in the marginal populations located in the area of Quiberon with an average of 0.51+/−0.04 and in the area of Locquirec (0.52+/−0.00) whereas central populations located in Roscoff and Porspoder showed higher values (respectively 0.61+/−0.01 and 0.67+/−0.02). Similarly, the allelic diversity (Na) and allelic richness (AR) were on average lower in marginal populations than in central ones.

The fixation index F_IS_, corresponding to the deficit of heterozygotes in a population, was relatively low for all populations studied, ranging from −0.019 in Sieck (Roscoff) to 0.085 in Molène (Porspoder). No clear difference was observed between marginal and central populations. Of the 396 individuals analyzed, 390 (98.4%) had unique seven-locus genotypes and 3 genotypes were repeated two times (6 individuals). These individuals with the same multilocus genotype were observed only in two sites in the marginal region of Quiberon (R not equal to 1, Table 1). The Psex values were all smaller than 0.05 (i.e., from 0.015 to 0.002) suggesting each repeated MLG was most likely produced by asexual reproduction.

### Production of spores and gametophytes

**Figure 3:**
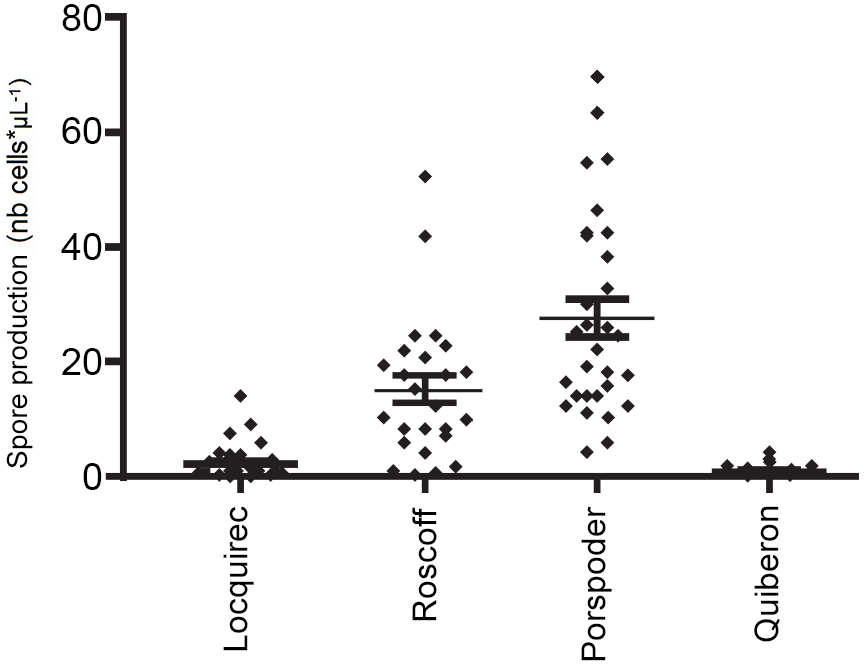
Spore release (number of spores per µL) by sporophytes of the four populations of *L. digitata*.

In initial trials we observed that gametophyte cultures were readily established from Roscoff sporophytes, but attempts often failed from sporophytes originating from Quiberon (data not shown). To investigate causes, spore production was examined from over 25 individuals from all four sites. Sporophyte individuals from Quiberon and Locquirec both showed greatly diminished spore release in comparison with individuals from Roscoff and Porspoder. Average spore production was 14-fold higher in Roscoff and 25-fold higher in Porspoder in comparison to Quiberon (Fig. 3).

### Spore ploidy

**Figure 4:**
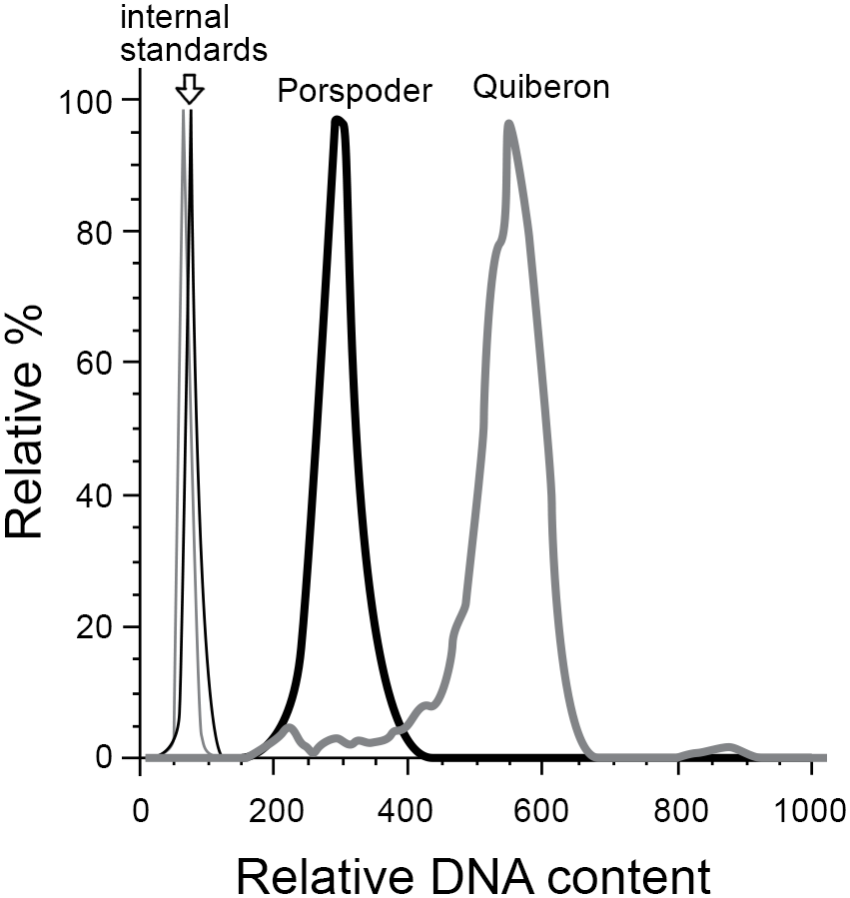
Example flow cytometric analysis of spore ploidy, showing histograms of Sybr Green I fluorescence (DNA content). Grey lines show a representative Quiberon sporophyte and black lines show a representative Porspoder sporophyte. In both cases the thick lines represent spores, and thin lines represent the internal standard (*Emiliania huxleyi* cells) added to the same sample during staining.

The spores produced by individuals from Roscoff, Porspoder, and Locquirec populations were predominantly haploid, whereas individuals from Quiberon produced high percentages of diploid spores (Table 2, Fig. 4). Microscopic observation of spores showed that diploid spores generally lacked flagella (data not shown).

**Table 2:** Flow cytometry results in the studied populations.

Population	N	2N	N & 2N	not scorable	Total
Roscoff	31/33	1/33	1/33	3	36
Porspoder	23/23	0/23	0/23	0	23
Quiberon	3/30	10/30	17/30	8	38
Locquirec	14/14	0/14	0/14	24	38

**N:** number of progenies displaying haploid profiles of DNA content. **2N:** number of progenies displaying diploid profiles of DNA content. **N & 2N:** number of progenies displaying haploid and diploid profiles of DNA content.

### Development, sex-ratio, and ploidy of gametophytes

The spores produced by the sporophyte individuals from sites in Quiberon germinated and developed normally into gametophytes. These gametophytes did not differ in color, shape or texture from gametophytes obtained from germination of spores from Roscoff individuals. In culture, these gametophytes initiated new juvenile sporophytes that for the first steps of development did not differ from those obtained from gametophytes of Roscoff.

Average sex ratios of the gametophytes obtained from spores of Quiberon were balanced (average of 0.461+/−0.185). However, three of the nine studied progenies displayed female bias, as judged by comparison to the binomial law (Table 3).

**Table 3:** Sex ratio in nine progenies of Quiberon.

Progeny	Total gametophytes in the progeny	Sex ratio
1	117	0.56
2	64	**0.33**
3	15	**0.13**
4	5	0.80
5	115	0.51
6	29	0.38
7	59	0.56
8	131	**0.40**
9	40	0.47

Bold numbers represent deviated sex ratios according to the binomial law.

**Figure 5:**
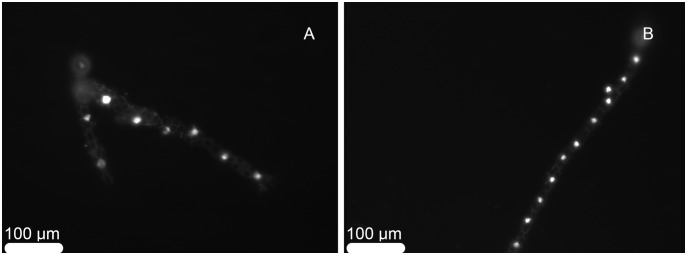
Gametophytes dyed with DNA stain. A) Female gametophyte from Quiberon. B) Female gametophyte from Roscoff.

**Figure 6:**
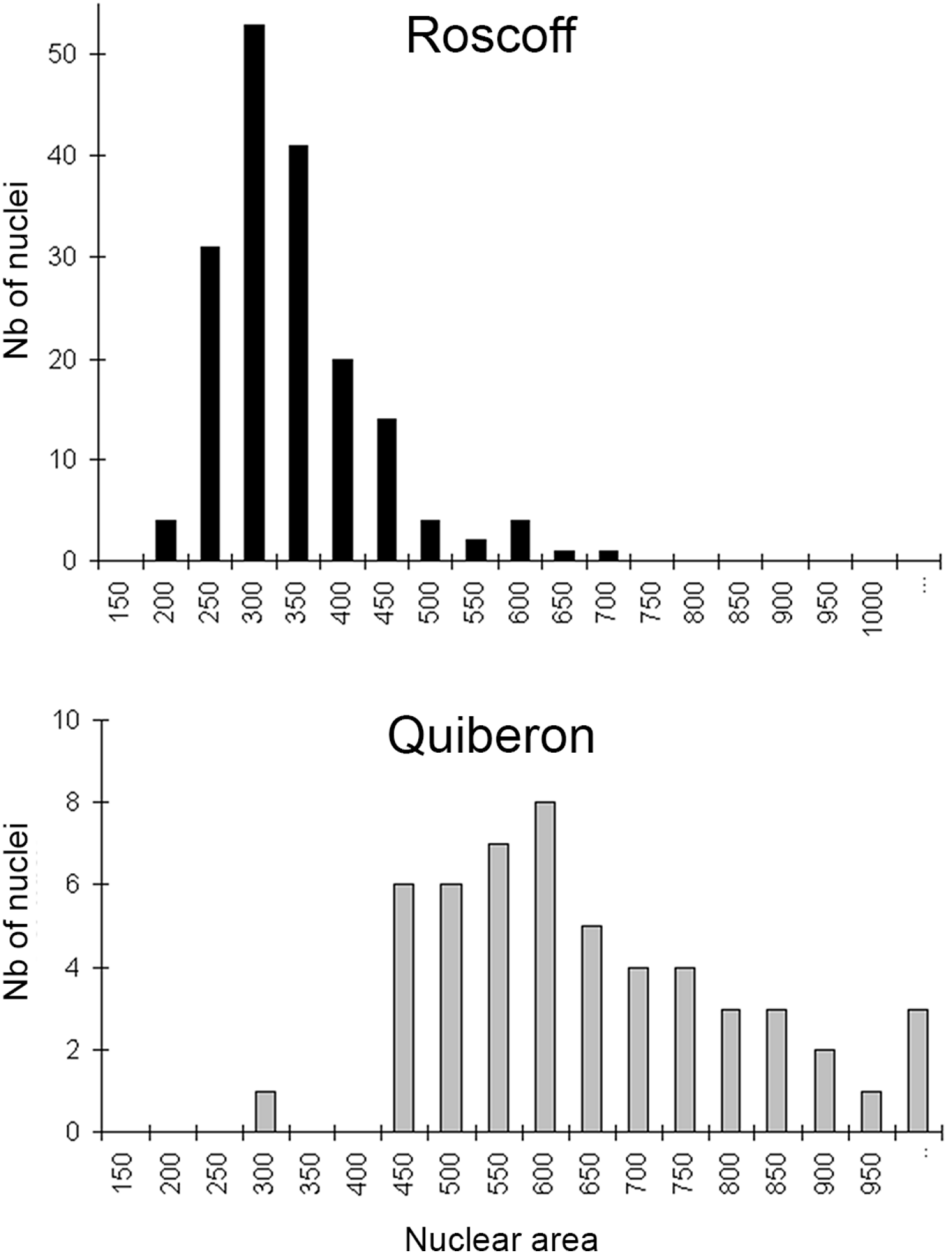
Nuclear area of gametophytes from central and marginal (Quiberon) populations.

The nuclei of filamentous gametophytes were visible under epifluorescence microscopy (Figure 5). The gametophytes obtained from spores of Quiberon displayed a significantly larger nuclear area than gametophytes of Roscoff (Kruskal-Wallis test: H = 104.44, dl = 1, p<0.0001) (Table S1, Figure 6).

The heterozygous sporophyte (Table S2) sampled in Roscoff produced gametophyte progeny which all showed only a single allele at each microsatellite locus (Ld371 and Ld531), and segregation was Mendelian. Most of the gametophyte progenies from heterozygous sporophyte individuals from Quiberon also showed only one allele at each locus (consistent with haploidy or homozygosity). However, out of all 121 gametophytes that were successfully genotyped at least at one locus, 9 gametophytes were detected that were heterozygous at least at one locus. Progenies from parents that were double heterozygotes are of particular interest, as they allow distinguishing between distinct types of apomeiosis and meiosis with automixis. Of 43 gametophyte progeny from four double-heterozygote parents where both loci successfully amplified, two gametophytes (4.7%) were double-heterozygote and two (4.7%) were single-heterozygote. The rest (90.7%) of gametophytes from which both loci amplified showed only one allele at both loci (see full results in Table S2, and example scoring of a heterozygous gametophyte in Fig. S1). Nuclear areas of gametophytes were also obtained from progenies of two of the Quiberon sporophyte parents included in Table S1 (parents 7 and 8, each of which was heterozygous at both loci). Although it was necessary to analyze nuclear area on the different individual gametophytes from those sacrificed for genotyping due to the limited amount of material from these microscopic stages, those progenies showed average nuclear areas ranging from 1.5–3.6 times (mean 2.3±0.6) the average nuclear areas of control Roscoff gametophyte progenies. 18 of the gametophytes in the progeny from these same parents were successfully genotyped at both loci, and one (5.6%) was heterozygous at only one locus, while of the six gametophytes that were only successfully genotyped at one locus, two were heterozygous (Table S1). Thus, gametophyte progenies that appeared to have predominantly diploid nuclear areas and arose from doubly-heterozygous parents mostly showed a single allele at each locus, suggesting they likely represent doubly homozygous diploids.

## Discussion

Of the four populations studied here, two populations could be considered as marginal based on genetic diversity analyses, but only one population was at the range limits defined by physiological tolerance (temperature effects on spore production). The Locquirec population was not near the geographic range limits of the species, yet this population is isolated from other populations by extensive sandy beaches. In contrast, the Quiberon population represents the southern-most limit of the range of the species distribution, and is exposed to more pronounced seasonal changes in temperature, with, in particular, a higher summer temperature that is frequently above the favorable temperature conditions in the field to produce meiospores [49], [50]. At the southern distributional limit of another algal species), Lee & Brinkhuis [51], reported that in August, while ripe sori were present, no meiospores were released. Recently, Bartsch et al. [50] demonstrated a clear inhibition of reproduction in *L. digitata* when temperature reaches 18°C, whereas reproduction is maximum at 10°C. Both the Quiberon and Locquirec populations showed reduced genetic diversity (He) and allelic richness (AR), consistent with lower immigration and diminished population size (confirming previous studies [34], [35], [36]. Additionally, both the Quiberon and Locquirec marginal populations also showed greatly reduced production of spores, yet only the Quiberon population showed an increase in the proportion of non-reduced spores (2N spores). This is consistent with previous evidence that there are two types of marginality for *Laminaria* species in Brittany: Geographical range-limit populations strongly affected by sub-optimal physical conditions versus populations that where fragmentation by distance may be the dominant force [52] (ecological rather than geographical range-limit). In the case of the Locquirec population, spore production was greatly diminished but the proportion of 2N spores produced was no higher than in individuals from the Roscoff population. Future studies would require sampling from multiple marginal populations of each type (range-limit marginality such as the Quiberon populations vs. isolation by unsuitable habitat at Locquirec), to determine if there is a consistent difference, with high proportions of non-reduced 2N spores only produced in range-limit marginal populations experiencing higher abiotic stresses. For the rest of the discussion, we therefore focus mostly on comparing the range-limit Quiberon population to the central populations (in Roscoff and Porspoder).

A variety of mechanisms for parthenogenesis or cloning have been demonstrated to be available to Laminarian algae in laboratory studies [27], [28], [31], [32], [53], [54]. Thus, we expected that *L. digitata* populations in Quiberon and/or Locquirec might also be displaying some form of geographical parthenogenesis, and that the parthenogenetic mechanism selected might help to distinguish between alternative hypotheses. For example, the production of spores by apomeiosis would preserve heterozygosity and locally adapted genotypes, while also permitting retention of a heteromorphic life cycle. Genetic data from the Quiberon populations in the field indeed detected individuals sharing the same MLGs produced by asexual reproduction at a low frequency (i.e. possibly produced by apomeiosis and/or fragmentation).

The nuclear area of male and female gametophytes developed from predominantly 2N spores of Quiberon individuals was consistent with gametophytes that are at least diploid. These diploid gametophytes are able to survive and to develop normally *in vitro.* Three mechanisms could allow diploid gametophytes: 1) Apomeiosis, the complete replacement of meiosis with mitotic divisions, would cause the production of gametophytes that were essentially genetically identical to the sporophyte parents, 2) Automixis, where two of the four nuclear products of meiosis fuse, resulting in diploid spores that were genetically different and, 3) Endomitosis, that would involve haploid spores restoring diploidy by chromosome doubling [55].

Although most of the gametophytes successfully cultured and genotyped from Quiberon *L. digitata* sporophyte parents exhibited only one allele at each locus, heterozygous gametophytes were detected, and two individuals showed a distinct diploid genotype than their parents (heterozygous at only one locus compared to parents that were heterozygous at both loci). As Quiberon parents typically produced some 1N spores (which would be expected to develop into normal haploid gametophytes), this result could be produced by a combination of 1N spores and 2N spores produced by automixis (with equal probability of fusion of all gametophyte products). However, nuclear area measurements suggested that the Quiberon gametophytes were apparently predominantly of diploid DNA content. This would imply that most progeny were homozygous diploids, with recombination causing heterozygosity in a small proportion. That would be consistent with failure specifically of Meiosis II after recombination between homologous chromosomes in Meiosis prophase, or automixis involving re-fusion preferentially of the two nuclear products of Meiosis II (terminal fusion). This mechanism, which has also been proposed in animals [56] and in the diatom *Thalassiosira angulata* [57] can result in diploid progeny that are predominantly homozygous but retain heterozygosity at some loci due to meiotic recombination between sister chromosomes (resulting in distinct chromatids attached to the same centromere and therefore co-segregating during Meiosis I). We caution that this particular mechanism is favored by the observation of small numbers of heterozygous gametophytes (with equal occurrences of double and single heterozygotes), as the ability to obtain gametophytes from Quiberon sporophytes was very limited and these heterozygous progeny were rare. The more important observation is that gametophytes produced from Quiberon sporophytes appeared to represent diploids that had lost most (if not all) of parent heterozygosity, so apomeiosis was not the dominant mechanism for formation of gametophytes.

Surprisingly, despite the apparent propensity for *L. digitata* to engage in parthenogenesis, microsatellite genetic data are not consistent with it being the major reproductive mode in natural populations. Automixis, especially terminal fusion automixis, would be expected to produce populations that are deficient in heterozygotes. Yet, although F_IS_ values for Quiberon were two times higher than in Roscoff, these values stayed close to zero, and so there was no important heterozygote deficiency. Endomitosis would lead to completely homozygous individuals and can also be ruled out as being important in the *L. digitata* natural populations examined here. Finally, the small number of repeated multi-locus genotypes detected suggests that parthenogenesis by apomeiosis might indeed be more frequent in Quiberon than in the central populations, but still remains rare.

Polyploidy is often associated with asexuality [9], [58] and may give ecological advantages in harsh environments, as is well documented for polyploidy plants [59], and also some animals [13]. An example is the study done on the ostracod *Eucypris virens* that reported that triploid, but not diploid asexual clones were able to colonize higher latitudes, expanding the range. It was suggested that the wider distribution range of triploids was due to elevated ploidy rather than asexuality [60]. It is still not clear if polyploidy itself or hybridization, which may include the generation of higher ploidies, promotes asexuality and further range expansion [61], [62], [63]. However in our study we did not find evidence of triploid or tetraploid sporophytes existing in natural populations of Quiberon. For each microsatellite locus analyzed, genotypes of the sporophytes were either homozygous or heterozygous, showing only 1 or 2 alleles and not higher numbers. Also, even individuals that produced predominantly diploid spores also typically produced at least a small proportion of spores with the normal haploid DNA content.

Marginality is usually associated with higher abiotic stresses, and the Quiberon habitat exhibits higher and more fluctuating regimes of temperature than central populations. As all kelps, *L. digitata* is sensitive to elevated temperature, and the observed change in its reproductive system might be an effect of exposure to higher and more fluctuating temperatures. The diminished spore production combined with the capacity for non-reduced spores to develop into gametophytes creates a greater proportional capacity for asexuality in marginal populations, similar to the pattern of geographical parthenogenesis frequently observed in terrestrial species. However, the production of high percentages of 2N spores by Quiberon individuals was not reflected in a restoration of total spore production capacity and gametophyte formation. Furthermore, population genetics shows no evidence that parthenogenesis is actually dominant in the maintenance of marginal populations of *L. digitata*.

Of the many hypotheses often invoked to explain the pattern of geographical parthenogenesis seen in terrestrial organisms, a dominant theme is that parthenogenic reproduction might provide particular genetic advantages either in avoiding inbreeding in small populations or in preserving well-adapted genotypes when dominant abiotic stressors are predictable, as opposed to biotic stressors which are highly variable and subject to co-evolution [13], [20], [61], [62]. Instead of asexuality being an adaptation advantageous in marginal populations, it seems that physiological factors first are specifically repressing sexual reproduction, and the low level intrinsic capacity for parthenogenesis (by various mechanisms in *Laminaria* species) does not increase enough to compensate. Such a maladaptive response at the southern edge of its distribution means that this European kelp species is at risk of local extinction as predicted by Ecological Niche Models under global change scenarios [64].

## Supporting Information

Figure S1 **Photo of an acrylamide gel that shows the genotyping of a sporophyte parent (lane 1) and three unambigously identifiable descendents including two homozygous gametophytes (lanes 2, 3) and one clear heterozygous gametophyte (lane 4).**
(DOC)

Table S1 **Nuclear area of gametophytes from Quiberon and Roscoff.**
(DOC)

Table S2 **Genotyping results for 8 progenies of Quiberon with two microsatellite loci.** Parents 1–8 are from Quiberon, parent 10 is from Roscoff. In each case the alleles are given for each locus in the parents, and the number of progeny with each combination of parental alleles is given (no progeny included alleles not found in the parent). Gametophytes where only a single allele was detected at each loci that was successfully scored might be haploid or homozygous diploids.
(DOC)
