# Supplementary material for: Alteration of Sexual Reproduction and Genetic Diversity in the Kelp Species Laminaria digitata at the Southern Limit of Its Range
Source: PLoS One. 2014 Jul 14;9(7):e102518. doi: 10.1371/journal.pone.0102518 (PMC4096927; doi:10.1371/journal.pone.0102518)
Supplement: Table S1 — Nuclear area of gametophytes from Quiberon and Roscoff. (DOC) [file pone.0102518.s002.doc]

|  | **Roscoff** |  |  |  |  | **Quiberon** | |  |  |  |  |  |
| --- | --- | --- | --- | --- | --- | --- | --- | --- | --- | --- | --- | --- |
|  | Male | Female | Male | Male | Female | Female | Male | Male | Male | Male | Male | Male |
| N° nuclei | Area | Area | Area | Area | Area | Area | Area | Area | Area | Area | Area | Area |
|  |  |  |  |  |  |  |  |  |  |  |  |  |
| 1 | 365 | 292 | 476 | 560 | 891 | 772 | 592 | 758 | 996 | 505 | 1100 | 488 |
| 2 | 374 | 396 | 487 |  | 791 | 810 | 735 | 1454 | 803 | 622 | 698 | 448 |
| 3 | 302 | 259 | 614 |  |  | 709 | 626 |  | 991 | 575 | 647 | 478 |
| 4 | 344 | 349 | 434 |  |  | 559 | 447 |  | 695 | 706 | 621 | 552 |
| 5 | 257 | 228 | 486 |  |  |  | 440 |  | 1030 | 652 | 705 | 577 |
| 6 | 397 | 209 | 460 |  |  |  | 542 |  |  | 543 | 975 | 677 |
| 7 | 310 | 307 | 405 |  |  |  | 512 |  |  | 820 | 870 | 402 |
| 8 | 243 | 244 | 516 |  |  |  | 543 |  |  | 949 |  | 810 |
| 9 | 377 | 280 | 296 |  |  |  | 571 |  |  |  |  | 541 |
| 10 | 282 | 293 |  |  |  |  | 585 |  |  |  |  | 674 |
| 11 | 427 | 273 |  |  |  |  |  |  |  |  |  |  |
| 12 | 244 | 275 |  |  |  |  |  |  |  |  |  |  |
| 13 | 303 | 312 |  |  |  |  |  |  |  |  |  |  |
| 14 | 242 | 302 |  |  |  |  |  |  |  |  |  |  |
| 15 | 444 | 433 |  |  |  |  |  |  |  |  |  |  |
| 16 | 327 | 426 |  |  |  |  |  |  |  |  |  |  |
| 17 | 295 |  |  |  |  |  |  |  |  |  |  |  |
| 18 | 268 |  |  |  |  |  |  |  |  |  |  |  |
| 19 | 279 |  |  |  |  |  |  |  |  |  |  |  |
| 20 | 212 |  |  |  |  |  |  |  |  |  |  |  |
|  |  |  |  |  |  |  |  |  |  |  |  |  |
| Mean | **314.6** | **304.88** | **463.8** | **560** | **841** | **712.5** | **559** | **1106** | **903** | **672** | **802** | **565** |
| Sd | 65.524 | 65.903 | 85.86 |  | 70.71 | 110.49 | 86.2 | 492 | 146 | 150 | 183 | 124 |

**Supplementary Table 1**
