# Supplementary material for: Alteration of Sexual Reproduction and Genetic Diversity in the Kelp Species Laminaria digitata at the Southern Limit of Its Range
Source: PLoS One. 2014 Jul 14;9(7):e102518. doi: 10.1371/journal.pone.0102518 (PMC4096927; doi:10.1371/journal.pone.0102518)
Supplement: Table S2 — Genotyping results for 8 progenies of Quiberon with two microsatellite loci. Parents 1–8 are from Quiberon, parent 10 is from Roscoff. In each case the alleles are given for each locus in the parents, and the number of progeny with each combination of parental alleles is given (no progeny included alleles not found in the parent). Gametophytes where only a single allele was detected at each loci that was successfully scored might be haploid or homozygous diploids. (DOC) [file pone.0102518.s003.doc]

**Supplementary Table 2**

| **Parent** | 1 | 3 | 5 | 6 | 7 | 8 | 9 | 10 |
| --- | --- | --- | --- | --- | --- | --- | --- | --- |
| Ld2-371 alleles: | 123/135 | 123/129 | 132/138 | 117/138 | 120/123 | 117/132 | 123/132 | 123/144 |
| Ld2-531 alleles: | 236/242 | 242/242 | 236/245 | 236/236 | 220/242 | 239/245 | 239/239 | 233/236 |
| Classification | Double Het | Single Het | Double Het | Single Het | Double Het | Double Het | Single Het | Double Het |
| **Progeny** |  |  |  |  |  |  |  |  |
| Hap or Double Hom | 14 | 4 | 8 | 16 | 12 | 5 | 15 | 5 |
| Single Het | 0 | 1 | 1 | 1 | 0 | 1 | 0 | 0 |
| Double Het | 1 | 0 | 1 | 0 | 0 | 0 | 0 | 0 |
| 1 locus, Hap o Hom | 5 | 5 | 7 | 5 | 2 | 2 | 5 | 2 |
| 1 locus, Het | 0 | 0 | 1 | 0 | 1 | 1 | 0 | 0 |
| both loci failed | 3 | 1 | 5 | 1 | 8 | 14 | 3 | 5 |
